# Hybrid Bladder Tumor: Urothelial Carcinoma With Squamous Cell Differentiation, Urothelial Sarcomatoid Carcinoma, and Concurrent Primary Mucinous Adenocarcinoma With Metastasis to the Penis

**DOI:** 10.7759/cureus.68894

**Published:** 2024-09-07

**Authors:** Petar Antonov, Gabriela Raycheva, Georgi Ivanov, Atanas Ivanov, Petar Uchikov, Veselin Popov, Georgi Tzigarovski, Alexandar Timev, Zhanet Grudeva

**Affiliations:** 1 Department of Urology and General Medicine, Medical University of Plovdiv, Plovdiv, BGR; 2 Department of Clinical Oncology, Medical University of Plovdiv, Plovdiv, BGR; 3 Department of General and Clinical Pathology, Medical University of Plovdiv, Plovdiv, BGR; 4 Department of Special Surgery, Medical University of Plovdiv, Plovdiv, BGR; 5 Department of Urology, Medical University of Sofia, Sofia, BGR

**Keywords:** local recurrence of bladder cancer, muscle-invasive bladder cancer, bladder adenocarcinoma, penile metastasis, hybrid bladder tumor

## Abstract

The most common histological variants of bladder cancer include urothelial, squamous, and adenocarcinoma. In high-grade, invasive urothelial carcinoma, divergent differentiation can be observed, including glandular, squamous, trophoblastic, and small-cell types. Urothelial sarcomatoid carcinoma is characteristic of advanced carcinomas and is considered a possible common end route for all epithelial carcinomas. Adenocarcinoma of the bladder refers exclusively to true glandular carcinomas. Hybrid tumors are extremely rare and consist of more than one tumor type within the total tumor mass. Penile metastases are extremely uncommon, and there are no reported cases of metastatic adenocarcinoma of the bladder in the literature.

## Introduction

Bladder cancer remains the most common malignancy of the urinary tract [1]. Its typical histological variants are urothelial, squamous cell, and glandular carcinoma. Urothelial muscle-invasive carcinomas often exhibit divergent differentiation into squamous, glandular, trophoblastic, and small cell types, occurring in approximately 30% of urothelial carcinoma cases [2]. Bladder adenocarcinomas are distinct from urothelial carcinomas with glandular differentiation, characterized by the formation of pseudocysts and glandular acini without columnar epithelium and mucin formation [3]. Secondary adenocarcinomas of the bladder are more common than primary ones, necessitating a comprehensive examination and differential diagnosis for the diagnosis of primary adenocarcinoma of the bladder. Sarcomatoid urothelial carcinomas are biphasic tumors composed of epithelial and mesenchymal components and are typically considered the advanced stage in the progression of all malignant epithelial neoplasms of the bladder. Hybrid neoplasms are exceedingly rare tumors consisting of two distinct tumor units, each corresponding to a specific tumor category, with both units originating within the same topographic area. Despite a well-established blood supply, penile metastases are extremely rare [4]. There are no reports in the literature of metastatic cases to the penis originating from primary adenocarcinoma of the bladder.

## Case presentation

**Figure 1:**
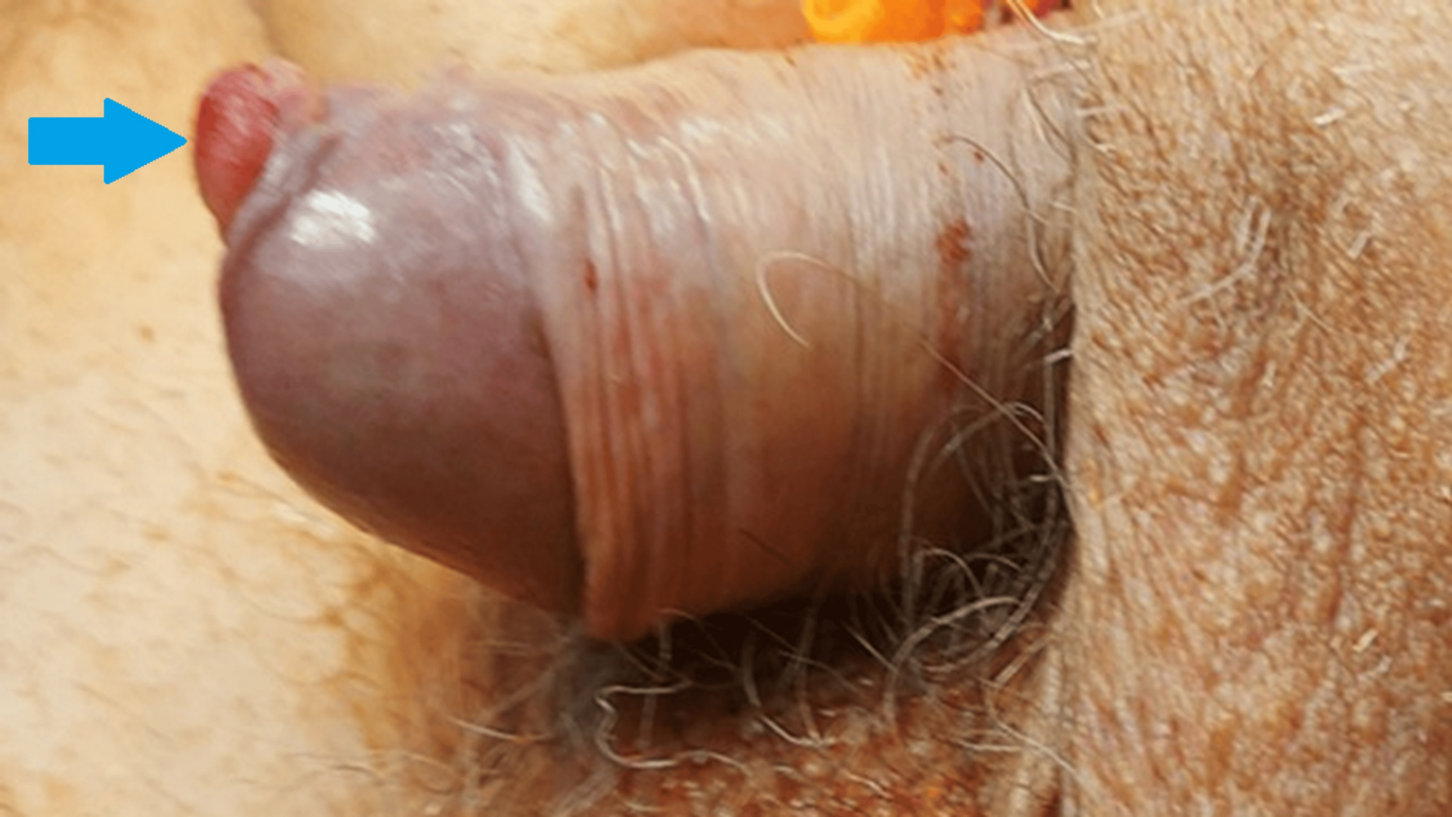
Lesion of the glans penis (blue arrow).

**Figure 2:**
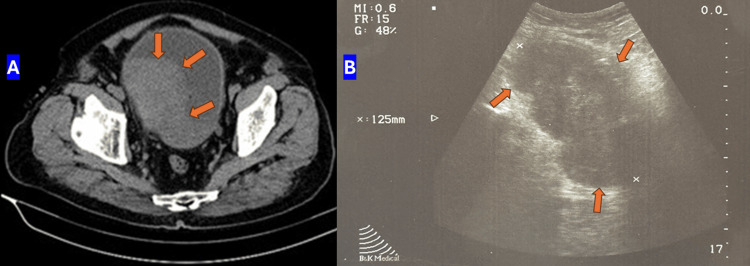
Tumor formation located in the bladder (orange arrows). A) Computed tomography of bladder; B) Ultrasound of bladder.

A 70-year-old man was hospitalized at the Urology Clinic due to macroscopic hematuria. He has a history of smoking, equivalent to over 40 pack years. Physical examination revealed a 1 cm tumor on the glans penis (Figure 1). Imaging examinations, including abdominal ultrasound and computed tomography (CT), confirmed hydronephrosis of the right kidney and a large bladder tumor (Figure 2).

**Figure 3:**
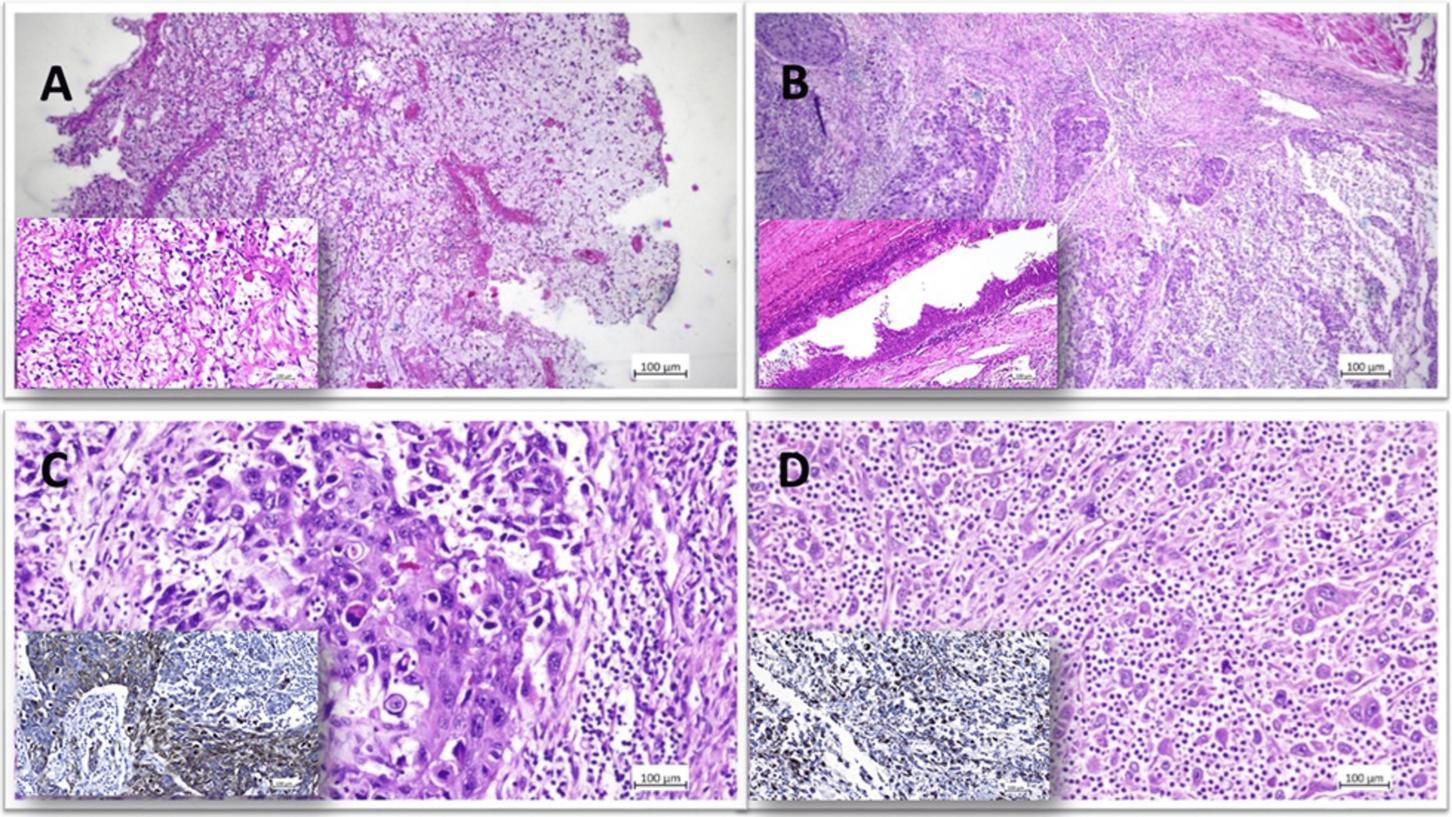
Hybrid bladder tumor. A) Mucinous adenocarcinoma HE x50 (inside mucinous adenocarcinoma HE x200); B) Urothelial carcinoma with squamous cell differentiation HE x50 (inside metaplasia of the urothelium HE x100); C) Urothelial carcinoma with squamous cell differentiation HE x200 (inside squamous cell differentiation PanCK x200); D) Sarcomatoid urothelial carcinoma HE x200 (inside a sarcomatoid component, vimentin x200).

Penile biopsy, cystoscopy with bladder biopsy, and placement of a nephrostomy tube on the right side were performed. Cystoscopy did not reveal pathological changes in the urethra or prostate gland. A tumor was identified in the bladder, occupying most of it with numerous clots. Subsequent histopathological examination of small tissue biopsies confirmed metastasis of mucinous adenocarcinoma to the penis and high-grade urothelial carcinoma in the bladder tissue, with areas showing primary mucinous features CK7 (+) in some cells, CK20 (-), AMACR (-), p63 (-), and squamous cell differentiation. Given the diagnosis of localized but muscular-invasive bladder disease, the patient underwent radical cystectomy with pelvic lymph node dissection, urethrectomy, and penile amputation. The urinary diversion was achieved with bilateral ureterocutaneostomy. Neoadjuvant chemotherapy was not administered due to severe macroscopic hematuria necessitating urgent cystectomy. Pathological examination of bladder samples revealed areas of sarcomatoid urothelial carcinoma, urothelial carcinoma with squamous cell differentiation, and primary mucinous adenocarcinoma invading the superficial muscular layer of the bladder (pT2a) (Figure 3).

**Figure 4:**
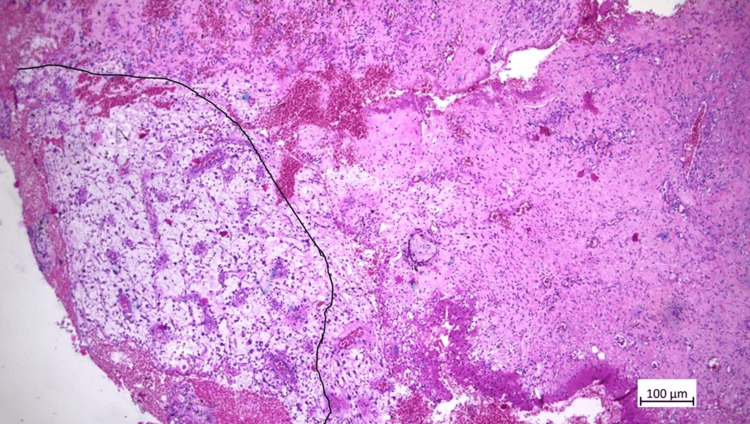
Metastatic focus of mucinous adenocarcinoma in the penis, around it with peritumoral edema and areas of hemorrhage.

The prostate gland, urethra, and lymph nodes were free of tumor infiltration, while mucinous adenocarcinoma infiltrated the corpus cavernosum of the penis (Figure 4). The patient was staged as pT2a N0 M1b.

**Figure 5:**
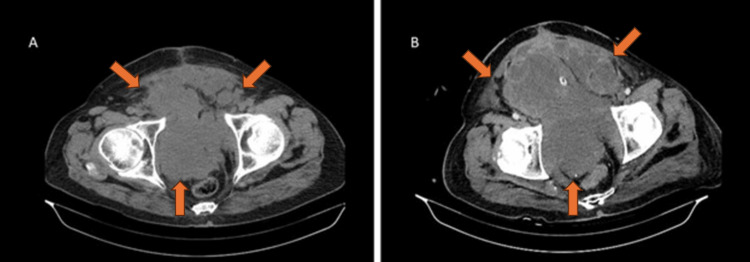
A) Local recurrence (orange arrows) - three weeks after radical cystectomy; B) Advanced local recurrence (orange arrows) - 1.5 months after radical cystectomy.

The early postoperative period was uneventful, but around the 15th day, a palpable mass at the surgical site and subsequent sparse bloody discharge prompted a pelvic CT scan (Figure 5A). Exploration revealed clots and a lymphocele. A follow-up CT scan (Figure 5B) indicated an increase in the mass size, leading to further surgical exploration revealing significant soft tissue formations in the pelvic region.

Histological examination confirmed infiltration of mucinous adenocarcinoma. The patient succumbed to uncontrollable bleeding from the wound drain and metabolic disorders due to cachexia, two months after the initial diagnosis.

## Discussion

Bladder cancer ranks fourth among all malignant diseases in men, following prostate, lung, and colorectal carcinoma [1]. The hallmark clinical symptom is painless macroscopic hematuria. Urothelial carcinomas constitute more than 90% of bladder cancers, while squamous cell carcinomas account for 5%, and adenocarcinomas and other histological variants make up less than 2%. In 2022, the World Health Organization (WHO) published the fifth edition of the classification of tumors of the urothelial tract, reflecting advancements in understanding the morphological diversity of urothelial carcinomas [2]. Urothelial carcinomas frequently demonstrate divergent differentiation, where alongside typical transitional cell carcinoma, morphological features of other types are observed. The incidence of invasive urothelial carcinomas with divergent differentiation in radical cystectomy specimens is substantial, reaching 33%. Squamous cell differentiation, characterized by intercellular bridges or keratinization, is found in up to 40% of invasive urothelial carcinoma cases [5]. There are no specific markers to separate squamous cell carcinoma differentiation from pure squamous cell carcinoma of the bladder [6], so the diagnosis is based on the clinical history and lack of or the presence of a clear conventional urothelial component in histological analysis. Glandular differentiation in urothelial carcinomas is the second most common variant, occurring in 6-18% of cases [7]. True glandular differentiation is defined by glands lined with columnar epithelium and mucin production, distinguishing it from pseudo glandular changes lacking cylindrical cells and mucin production [3]. Histological analysis of our patient's biopsies indicated adenocarcinoma morphology, specifically the characteristics of true glandular tumors rather than those with divergent glandular differentiation. Adenocarcinoma of the bladder is a rare and aggressive neoplasm originating from urothelial cells, presenting with a predominantly glandular phenotype [8]. It can be primary or secondary, with secondary adenocarcinomas more common and originating from various primary sites including the colon, prostate, endometrium, cervix, breast, and lungs.

Diagnosis of primary bladder adenocarcinoma requires the exclusion of metastasis from other organs [9]. Therapeutically, adenocarcinomas of the bladder are categorized into urachal and non-urachal types, with histological subtypes including enteric, mucinous, clear cell, ring cell, hepatoid, unspecified adenocarcinoma, and mixed forms. Retrospective studies generally indicate a poorer prognosis for adenocarcinoma compared to urothelial carcinoma [10], as observed in our case due to aggressive disease progression including penile metastasis and local recurrence. Sarcomatoid urothelial carcinoma of the bladder is frequently associated with advanced disease and a dismal prognosis [11]. This subtype is characterized by the presence of both epithelial and mesenchymal components, typically high-grade spindle cell sarcoma, with vimentin used as a supportive marker. It is suggested that sarcomatoid carcinoma may represent a common end stage of epithelial bladder tumors [12]. Local recurrence after radical cystectomy commonly involves soft tissues of the pelvis or pelvic lymph nodes, attributed to incomplete tumor excision, positive surgical margins, inadequate lymph node dissection, or intraoperative tumor cell spillage [13]. Typically detected 2-3 years post-surgery [14], our patient experienced rapid and fatal local recurrence within two months of initial treatment. Imaging studies for invasive aggressive bladder carcinomas are multifaceted and include a combination of CT, MRI, PET-CT, and other methods, depending on the patient's clinical situation. The choice of appropriate investigations depends on the stage of the disease, symptoms, and the patient's overall condition, as well as the presence of local spread and metastases. Adjuvant treatment for aggressive histology types of bladder carcinoma is a multidisciplinary approach that typically includes a combination of chemotherapy, radiotherapy, and, in some cases, targeted therapy or immunotherapy, depending on the patient's individual characteristics and the stage of the disease. The decision for a specific treatment regimen should be made in consultation with an oncology team to optimize the therapeutic strategy. Despite the ample blood supply to the penis, penile metastases are rare and often indicate a poor prognosis [4]. Various mechanisms of metastasis have been proposed, including direct tumor spread, retrograde venous spread, arterial spread, lymphatic spread, and instrumental spread. The first description of penile metastasis by Eberth dates back to 1870 [15], and since then, 144 cases of penile metastasis from bladder cancer have been documented. These cases predominantly involve primary urothelial carcinoma, often with squamous cell differentiation. Adenocarcinomas that metastasize to the penis typically originate from the prostate gland or colon [16]. The hybrid carcinoma presented here represents the first documented case of metastatic adenocarcinoma in the penis. Our case is unique in that it involves a synchronous diagnosis of primary bladder adenocarcinoma and metastasis to the penis, contrasting with the metachronous nature typically observed in previously reported cases of urothelial bladder carcinoma [17].

## Conclusions

Hybrid neoplasms are casuistically rare tumors consisting of two distinct tumor units, each corresponding to a specific tumor category, with both units originating within the same topographic area. Divergent morphology is frequently encountered in high-grade urothelial carcinomas. The hybrid bladder carcinoma described in this case encompasses primary adenocarcinoma, sarcomatoid urothelial carcinoma, and urothelial carcinoma with squamous cell differentiation, culminating in metastasis to the penis and aggressive local recurrence of the adenocarcinoma component.
